# Associations of CSF Angiogenic Factors with Cerebrovascular Lesion in Alzheimer’s Disease

**DOI:** 10.1002/alz.086409

**Published:** 2025-01-03

**Authors:** Jun Lin, Jiong Shi

**Affiliations:** ^1^ University of Science and Technology of China, Hefei, Hefei China; ^2^ The First Affiliated Hospital of USTC, Neurology Department, Hefei China

## Abstract

**Background:**

Mounting evidence suggests an increase in angiogenesis in the Alzheimer’s disease (AD) brain, but the role of this process in AD remains uncertain. The present study aims to explore the association between CSF angiogenic factors that promote angiogenesis and cerebrovascular lesions as well as neurodegeneration.

**Method:**

The cross‐sectional study included 104 individuals with a CDR score of ≤ 0.5 and 96 individuals with a CDR score > 0.5. We measured 12 angiogenic factors using immunoassays. Cerebrovascular lesions were assessed through CSF PDGFRβ, Qalb, and WMH volume, while neurodegeneration was evaluated by cortical thickness and serum NFL. Cognitive function was measured using MMSE.

**Result:**

Higher tau pathology showed associations with increased levels of CSF sVEGFRII, VEGF‐C, VEGF‐D, PLGF, Angiopoietin2, and Serpin E1. Although both Aβ pathology and tau pathology were associated with elevated levels of CSF sVEGFRI, sTIE2, and bFGF, the effects of Aβ pathology seemed to be influenced by tau pathology. Moreover, higher levels of sVEGFRII, sTIE2, PLGF, Serpin E1, IL8, and lower levels of VEGF‐C and uPA were linked to increased CSF PDGFRβ, Qalb, or WMH volume. Furthermore, higher levels of sVEGFRI, sTIE2, PLGF, Angiopoietin2, IL8, and lower levels of VEGF‐A and uPA were associated with reduced cortical thickness, increased serum NFL, and decreased MMSE scores.

**Conclusion:**

Tau pathology may play a critical role in regulating the elevated CSF angiogenic factors, which could contribute to cerebrovascular lesions and neurodegeneration in AD. These results highlight the therapeutic potential of inhibiting hypervascularization or promoting the maturation of neonatal blood vessels in AD.

